# Complication with Removal of a Lumbar Spinal Locking Plate

**DOI:** 10.1155/2015/787249

**Published:** 2015-03-09

**Authors:** Brooke Crawford, Christopher Lenarz, J. Tracy Watson, Dirk Alander

**Affiliations:** Department of Orthopaedic Surgery, Saint Louis University, 3635 Vista Avenue, St. Louis, MO, USA

## Abstract

*Introduction*. The use of locking plate technology for anterior lumbar spinal fusion has increased stability of the vertebral fusion mass over traditional nonconstrained screw and plate systems. This case report outlines a complication due to the use of this construct. *Case*. A patient with a history of L2 corpectomy and anterior spinal fusion presented with discitis at the L4/5 level and underwent an anterior lumbar interbody fusion (ALIF) supplemented with a locking plate placed anterolaterally for stability. Fifteen months after the ALIF procedure, he returned with a hardware infection. He underwent debridement of the infection site and removal of hardware. *Results*. Once hardware was exposed, removal of the locking plate screws was only successful in one out of four screws using a reverse thread screw removal device. Three of the reverse thread screw removal devices broke in attempt to remove the subsequent screws. A metal cutting drill was then used to break hoop stresses associated with the locking device and the plate was removed. *Conclusion*. Anterior locking plates add significant stability to an anterior spinal fusion mass. However, removal of this hardware can be complicated by the inherent properties of the design with significant risk of major vascular injury.

## 1. Introduction

Successful techniques in limb fracture fixation and deformity correction are often expanded to other areas of the body. For plate fixation, these techniques have evolved from nonconstrained devices to the use of fixed or variable angle locking plate and screw constructs. The development of the LISS locking plate in the mid-1990s and the subsequent evolution of locked plating devices advanced care of extremity injuries by providing very stable constructs for challenging extremity fractures [1–3]. As the orthopaedic community began to understand the advantages of locked plating, its application to other surgical sites and clinical conditions expanded including the development of an anterior locking plate for spine fusions.

The push for less invasive and muscle sparing approaches to the spine, along with efforts to save time while providing a stable fusion environment, gave impetus for the development of anterior plates for use in lumbar spine fusions. Stability of nonconstrained screw and plate systems for the lumbar spine is primarily in extension and does not provide significant stability for coronal and rotational moment arms. Utilizing locked plate technology creates a fixed angle construct that provides a strong constraint in all three planes of motion [4–8]. The fixed screws and cold weld between the screw head threads and the plate minimize the potential for loosening and migration, especially in osteoporotic bone. Problems associated with locking plates in the anterior spine include difficulty with bending large plates because of their large size, plate thickness, the location under great vessels, and cost. This case report identifies another concern with anterior spinal locking plates: difficulty of removal in a well-fixed plate in the lumbar spine and the associated potential for vascular disaster.

## 2. Case

**Figure 1:**
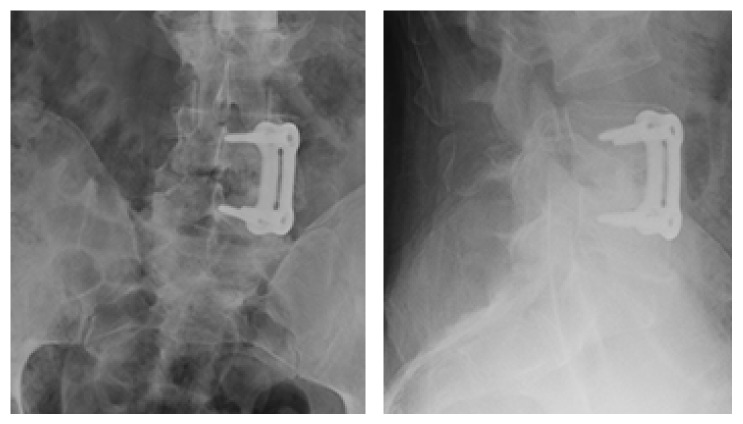
AP and lateral radiographs of lower lumber spine showing the anterolateral locking plate at L4-5 (Synthes 4.5 mm anterolateral spinal locking plate, with 5.5 mm titanium locking screws, West Chester, Pennsylvania, United States).

A 44-year-old male involved in a motor vehicle collision presented with polytrauma that included L2 burst fracture. He underwent a corpectomy of L2, anterior vertebral reconstruction, and fusion utilizing a carbon-fiber cage and lateral spinal instrumentation (Kaneda device). Two months after his surgery, he developed increasing back pain and was found to have pyogenic discitis in his previously degenerated L4/5 disc between his fusion levels of L1 through L3 and his previous L5 to sacrum fusion. He underwent extensive debridement of the L4/5 disc and paraspinal tissues, as well as an L4/5 anterior discectomy and fusion with femoral allograft secured with a Synthes Spine titanium anterolateral locking plate (Figure 1). Cultures taken at the time of this surgery indicated a methicillin sensitive staphylococcus aureus infection, and postoperatively the patient was treated with intravenous Ancef, 1 gram every 8 hours for a total of six weeks.

One year after his L4/5 fusion, the patient developed increasing pain in his lower back along with laboratory studies consistent with recurrent infection. Radiographic studies indicated changes at the L1–3 fusion mass, indicating the infection may not be isolated to the previous L4/5 disc. We planned a staged surgical debridement, hardware removal, and reconstruction, first at the L1–3 site, followed by debridement, hardware removal, and reconstruction at the L4-5 site. Exploration of the L2 corpectomy site confirmed a pyogenic infection extending into the psoas. After this was debrided aggressively, we reconstructed the L1–3 space with tibial allograft, followed by posterior segmental instrumentation. Cultures again grew methicillin sensitive staph aureus, and the patient was placed on IV vancomycin and gentamicin. Ten days later, a paramedian approach was used to access to the L4/5 locking plate, where purulence was again noted. Using the standard screw driver we were unsuccessful in our attempt to remove any of the screws and stripped the screw interfaces. We were successful in removing one screw with a reverse threaded screw driver, but we subsequently broke a total of 3 reverse threaded drivers attempting to remove the final three screws (Figure 3).

**Figure 2:**
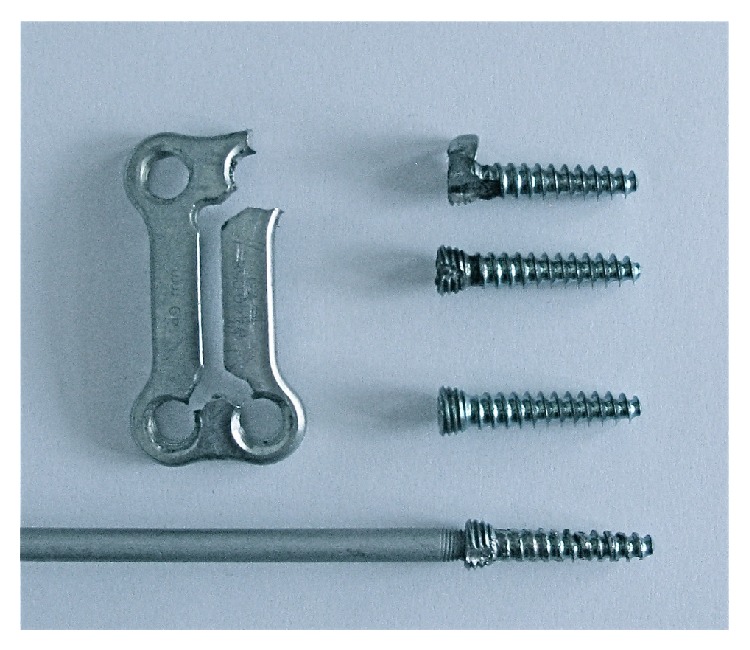
Photograph of the plate after removal, demonstrating the cold welding of the screw head to the plate (top screw: this was the one release with a peripheral burr cut, top right hole of plate), the trajectory of the burr holes made to release the plate (middle two screws, requiring burr cuts from the center of the plate to each hole, bottom two holes), and the cold weld of the reverse threaded screw driver to the screw head (bottom screw, top left hole).

**Figure 3:**
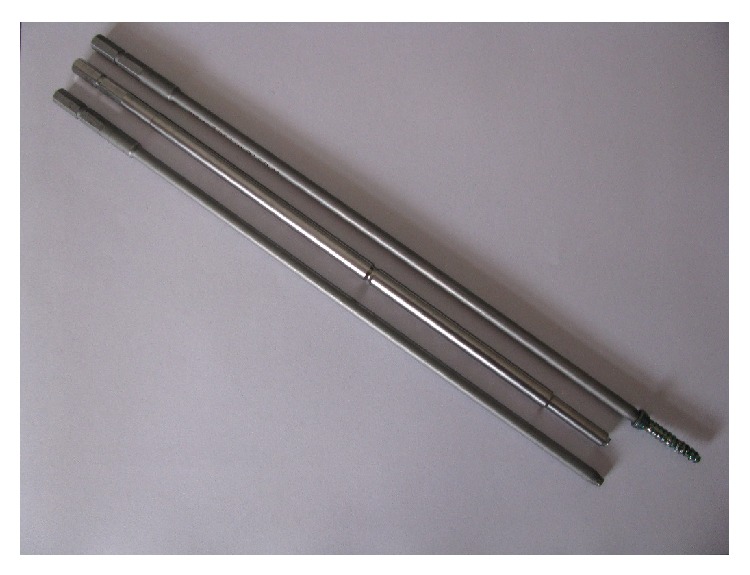
Image of the three reverse threaded screw drivers used. The top driver was successful in removing the screw but cold-welded to the interface. The bottom two drivers broke distally when used unsuccessfully on the remaining screws.

Complete hardware removal was considered essential to treat this patient's infection. To protect the great vessels, they were mobilized and retracted medially as much as possible. A combination of various sized metal malleable retractors was secured between the vessel and area of burring. A high speed burr (Stryker TPS) was used with both an aggressive 4 mm round tip burr and a 3 mm neuro tipped cutting burr. Each tip was replaced frequently before they became dull. Cuts were started inside the plate and directed towards the screw head to break the screw plate hoop stresses. This inside-out technique avoided the vessel walls which were in contact with the outer edges of the plate. The final screw was drilled from the periphery to the screw head because it was located farthest away from the vessels with adequate space available for the drill bit. Once the plate threads were released the screw was easily removed (Figure 2). The L4/5 fusion was solid with no signs of pseudoarthrosis, so no further stabilization was utilized. Final cultures continued to grow the same MSSA, and postoperatively we initiated a 6-week course of nafcillin, 2 grams every 4 hours. The patient is now over eight years out from his final operation without any recurrences of infection and with great improvement in his pain.

## 3. Discussion

The difficulties encountered with the removal of locked plates in orthopaedic traumatology are well recognized. These problems have been universally reported to be with titanium implants usually due to the phenomenon of cold welding of the locking screw heads into the locking screw holes. Other problems reported with the removal of titanium locking implants are related to the relative malleability of the titanium screw heads. As the torque required to remove a jammed (cold welded) screw increases, often the screw head fails to maintain a competent interference fit with the screw driver. This can result in many potential problems including damage to the recesses (stripping) of the screw head or the screwdriver, jammed screws, broken screws, and shearing off the screw head due to rigid interface of bone ingrown into the screw threads.

Another proposed mechanism for the difficulty of locked screw removal is related to the eccentric placement of the screw head into the locking screw hole, producing a cross thread mechanical lock which is not necessarily due to cold welding. Again, with the more malleable titanium implants, this phenomenon of cross threading is more likely to occur in an unrecognized fashion compared to a stainless implant, especially if the screw placement is off axis (nonorthogonal) with regard to the screw hole. Most of these removal issues have* not* been reported when stainless steel implants have been utilized.

Many techniques have been described to overcome these difficulties. Most frequently, in the case of a cold welded screw, the technique of radial side cut into the screw hole releases the hoop stresses about the screw [9]. Other techniques described for removing locking screws use specific instruments including hollow reamers, conical extraction screws, extraction bolts, and carbide drill bits.

There are difficulties encountered when using a high speed burr to perform a radial side cut to burr down a screw head. Heat generation with subsequent bone and tissue necrosis may be an issue, especially when removing these devices around sensitive structures, such as nerves and vessels. The issue of metal debris is also a concern, especially in certain locations. Debris can be collected easily by spreading a sterile, water soluble gel (Doppler gel) to cover local soft tissues. The debris is then captured in the gel and held locally, allowing for easy removal at the end of the procedure.

For the spine specifically, anterior lumbar plating with locking screw plate interfaces allow for the use of fixed angle constructs, improving stability and rigidity in all planes of motion [4–8]. When used in conjunction with anterior interbody fusion, this construct provides excellent three-dimensional stability and obviates the need for posterior fixation. When removal of this device is necessary, however, the inherent design can lead to significant difficulties and can have potentially catastrophic complications. Techniques for removal of this type of device have not been reported in the spine literature. In review of extremity trauma literature, use of metal cutting burrs and wheels to remove a locked plate from the bone is often necessary [2, 10–12]. Normally, in extremity injuries, these plates are in locations on the long bone which do not place large vascular and neurologic structures at significant risk [13]. In the lumbar spine there is limited access to the plate and its location due to close proximity of the great vessels.

In situations where an anterior spinal locking plate requires removal from the lumbar spine, we recommend the following techniques based on the described case: first, expect that cold welding of the screws may have occurred when the plate was placed and have a high speed burr ready with several drill bits in anticipation of having to drill through the plate. The great vessels will be in close proximity, including the aortic bifurcation around the level of L4, and the confluence of the vena cava at L5. An approach surgeon who is very comfortable with vascular surgery can assist with vessel mobilization and with any complications that may arise with the vessels. Malleable retractors can be used to lightly retract, but more to protect the vessels; they can simply be anchored on the bone between the vessel and the area to be drilled. Finally, a sterile ultrasound jelly or lube can be applied around the area to be drilled to catch the metal fragments so they are easier to remove. By anticipating the complication of cold welding that can require hardware drilling and the potential associated complication of vascular injury, these precautions should allow for a safe patient outcome.
